# Draft Genome Sequence of a Benzothiophene-Desulfurizing Bacterium, *Gordona terrae* Strain C-6

**DOI:** 10.1128/genomeA.00381-13

**Published:** 2013-06-20

**Authors:** Wei Wang, Ting Ma, Yi Ren, Guoqiang Li

**Affiliations:** Key Laboratory of Molecular Microbiology and Technology, Ministry of Education, TEDA School of Biological Sciences and Biotechnology, Nankai University, TEDA, Tianjin, Chinaa; Tianjin Key Laboratory of Microbial Functional Genomics, Tianjin, Chinab; College of Life Sciences, Nankai University, Tianjin, Chinac; School of Food Engineering and Biological Technology, Tianjin University of Science and Technology, Tianjin, Chinad

## Abstract

*Gordona terrae* strain C-6 was isolated from oil-contaminated soil and is capable of desulfurizing benzothiophene (BT). Here we report the draft genome sequence of *G. terrae* strain C-6, which may help to reveal the genetic basis of the BT biodesulfurization pathway.

## GENOME ANNOUNCEMENT

*Gordona terrae* strain C-6 is a benzothiophene-desulfurizing bacterium that was isolated from oil-contaminated soil of the GuDao Oil Field, China. It can desulfurize benzothiophene (BT) exclusively but not dibenzothiophene (DBT) to yield *o*-hydroxystyrene as the final product (1). To date, two major DBT biodesulfurization pathways (“Kodama” and “4S”) have been reported, and the functions of genes encoding them have been fully described as well (2–4). In contrast, the genetic basis of BT metabolism still remains unclear, except that only two types of BT biodesulfurization pathways were proposed (5, 6). To promote better understanding of genetic information regarding the BT biodesulfurization pathway, we herein report the draft genome sequence of *G. terrae* strain C-6.

The whole-genome sequencing was performed using the Illumina HiSeq 2000 platform at the Majorbio Bio-Pharm Technology Co., Ltd., Shanghai, China. *De novo* assembly was performed by using SOAPdenovo (http://soap.genomics.org.cn/soapdenovo.html). Gene prediction and annotation were carried out using the NCBI Prokaryotic Genome Annotation Pipeline (http://www.ncbi.nlm.nih.gov/genomes/static/Pipeline.html). The metabolic pathways were additionally annotated using the Kyoto Encyclopedia of Genes and Genomes (KEGG) database (7).

We generated a total of 28,223,994 filtered paired-end reads, with a mean length of 101 bp, corresponding to approximately 600-fold coverage of the genome. A total of 130 contigs were generated, with an N_50_ of 125,123 bp. Eighty-seven of the contigs were >1 kb in length, and the largest contig measured 376,011 bp. The final genome draft consisted of 5,170,241 bp, with a G+C content of 67.9%. In total, 4,645 protein-coding genes (protein-coding sequences [CDS]) and 46 tRNAs were predicted. Among the CDS, 3,149 and 1,730 proteins could be assigned to COG families and KEGG orthologous groups, respectively.

According to the reported information of known DBT and proposed BT biodesulfurization pathways, a number of genes possibly involved in BT biodesulfurization were predicted from the C-6 genome based on gene annotation. Among these were 5 genes that encode FMNH_2_-dependent monooxygenase and 5 genes that encode alkanesulfonate monooxygenase, which were proposed to be responsible for the first three steps in BT biodesulfurization (5, 6, 8), and some genes which may be responsible for the transport of BT and its metabolites. Most of the predicted genes involved with BT biodesulfurization were in four clusters and located in contig 1, 5, and 18. Only one gene cluster (in contig 1) contained a putative desulfinase gene, which catalyzes the last step of the 4S desulfurization pathway and hydrolyzes sulfinate or sulfonate from benzene sulfinate or benzene sulfonate (9). This gene cluster is therefore thought to be the most likely to be responsible for the BT desulfurization pathway.

### Nucleotide sequence accession numbers.

The whole-genome shotgun project of *G. terrae* strain C-6 has been deposited at DDBJ/EMBL/GenBank under the accession no. AQPW00000000. The version described in this paper is the first version, no. AQPW01000000.
